# Regulation of plasma histamine levels by the mast cell clock and its modulation by stress

**DOI:** 10.1038/srep39934

**Published:** 2017-01-11

**Authors:** Yuki Nakamura, Kayoko Ishimaru, Shigenobu Shibata, Atsuhito Nakao

**Affiliations:** 1 Department of Immunology, Faculty of Medicine, University of Yamanashi, 1110 Shimokato, Chuo, Yamanashi 409-3898, Japan; 2 Department of Physiology and Pharmacology, School of Advanced Science and Engineering, Waseda University, 2-2, Wakamatsu-cho, Shinjuku-ku, Tokyo, 162-8480, Japan; 3 Atopy Research Center, Juntendo University School of Medicine, 2-1-1 Hongo, Bunkyo-ku, Tokyo, 113-8421, Japan

## Abstract

At steady state, plasma histamine levels exhibit circadian variations with nocturnal peaks, which is implicated in the nighttime exacerbation of allergic symptoms. However, the regulatory mechanisms are largely unexplored. This study determined how steady-state plasma histamine levels are regulated and affected by environmental factors. We found that plasma histamine levels decreased in mast cell–deficient mice and their circadian variations were lost in mast cell–deficient mice reconstituted with bone marrow–derived mast cells (BMMCs) harboring a mutation in the circadian gene *Clock. Clock* temporally regulates expression of organic cation transporter 3 (OCT3), which is involved in histamine transport, in mast cells; OCT inhibition abolished circadian variations in plasma histamine levels. Mice housed under aberrant light/dark conditions or suffering from restraint stress exhibited de-synchronization of the mast cell clockwork, concomitant with the loss of circadian variations in OCT3 expression and plasma histamine levels. The degree of compound 48/80–induced plasma extravasation in mice was correlated with plasma histamine levels. Collectively, the mast cell clock mediates circadian regulation of plasma histamine levels at steady state, in part by controlling OCT3 expression, which can be modulated by stress. Additionally, we propose that plasma histamine levels potentiate mast cell–mediated allergic reactions.

Histamine is a biogenic amine that plays an important role in inflammatory responses, in particular, mast cell-mediated allergic reaction^1^,^2^. Histamine in the body is generated and stored in granules in mast cells, basophils and eosinophils^3^. But, non-immune cell histamine is found in several tissues, including the brain and the enterochromaffin-like (ECL) cell of the stomach, where it functions as a neurotransmitter and a stimulant for gastric acid release, respectively^3^. It has been extensively studied how histamine release occurs when mast cells are activated^4^. However, it remains poorly understood how tissue or plasma histamine levels are regulated under normal conditions and what the precise physiological/pathological significance of this phenomenon is.

It has been documented as early as 1960’s that base-line plasma histamine levels exhibit circadian variations in rodents and humans^5^,^6^,^7^,^8^,^9^,^10^. For example, in the circadian pattern of plasma histamine concentrations in rats, peak levels occur at the end of the light phase, *i.e*., at the end of the resting phase for nocturnal rodents^6^. In humans, plasma histamine levels increase in the early hours of the morning in healthy volunteers or asthmatic patients^7^,^8^,^9^,^10^. Although still controversial^10^, these nocturnal peaks in steady-state plasma histamine levels are implicated in the nighttime exacerbation of asthma symptoms^7^,^9^. In any case, there has been little information about the precise mechanisms that regulate plasma histamine levels at steady state.

The circadian clock drives daily oscillations of behavior and physiology, such as sleep–wake cycles and hormonal secretion^11^,^12^,^13^. In mammals, the circadian clock system consists of the central oscillator, located in the suprachiasmatic nucleus (SCN) of the hypothalamus, and peripheral oscillators in virtually all cell types, including mast cells. Light is a key zeitgeber (a German term meaning time giver, which describes a variable capable of phase-setting the circadian clock), which transmits the environmental light information to the central SCN clock via the retinohypothalamic tract and sets the rhythm of the central clock. The central SCN clock, in turn, passes this information on to peripheral clocks via neural and endocrine pathways. Circadian rhythmicity in individual cells is created by a series of oscillating clock proteins that are involved in a transcriptional–translational feedback loop (TTFL)^11^,^12^,^13^. Briefly, CLOCK and BMAL1 heterodimerize and activate transcription of *Period (Per)1*/*2* (PER), *Cryptochrome (Cry)1*/*2* (CRY), and other clock controlled genes (CCGs) via E-box or E-box–like elements in their promoter regions. The CRY and PER proteins, after a time lag, enter the nucleus and inhibit their own transcription in different manners. CRY binds to the CLOCK/BMAL1-E-box complex and inhibits transcription whereas PER represses the CLOCK/BMAL1-E-box complex–mediated transcription by removing the heterotrimeric complexes in a CRY–dependent manner^14^,^15^,^16^. With several other post-transcriptional modifications, this core TTLF loop generates ~24-hour oscillations in the expression of “clock genes” and CCGs in individual cells and maintains rhythmicity at the cellular and organismal levels.

In this study, we investigated whether mast cells are predominantly responsible for maintenance of baseline plasma histamine levels in mice. We then investigated whether the mast cell–intrinsic clock plays a role in the circadian regulation of plasma histamine levels at steady state, and, if so, how this mast cell clock–dependent mechanism is affected by environmental factors.

## Results

### The mast cell clock mediates circadian regulation of plasma histamine levels in mice

**Figure 1:**
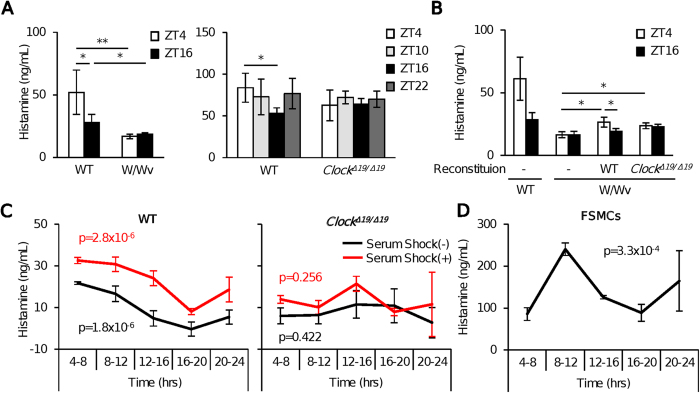
The mast cell clock mediates temporal regulation of plasma histamine levels in mice. (**A)** Comparisons of steady-state plasma histamine levels at the indicated time points between wild-type and mast cell–deficient mice (left panel), and between wild-type mice and *Clock*^*Δ19*/*Δ19*^ mice (right panel). (n = 5) (One-way ANOVA with Bonferroni’s test). (**B)** Plasma histamine levels at ZT4 or ZT16 in the steady states in mast cell–deficient mice reconstituted with subcutaneous injections of wild-type BMMCs or *Clock*^*Δ19*/*Δ19*^ BMMCs. (n = 5–7) (One-way ANOVA with Bonferroni’s test). (**C)** Kinetics of net histamine release from wild-type and *Clock*^*Δ19*/*Δ19*^ BMMCs during the indicated culture periods, with or without serum shock. (n = 4) (One-way ANOVA). (**D**) Kinetics of net histamine release from fetal skin–derived mast cells (FSMCs) during the indicated culture periods. (n = 4) (One-way ANOVA) Similar results (**A–D**) were obtained at least from 2 independent experiments. The values represent means ± SD, *p < 0.05, **p < 0.01.

We first investigated whether mast cells play a role in the maintenance and circadian regulation of steady-state plasma histamine levels in mice. Plasma histamine levels fell to ~70% in mast cell–deficient W/Wv mice compared with those in control mice (Fig. 1A). The time-of-day–dependent variation in plasma histamine levels observed in wild-type mice was also absent in mast cell–deficient W/Wv mice (Fig. 1A). Reconstitution of mast cell–deficient W/Wv mice with wild-type bone marrow–derived mast cells (BMMCs) increased plasma histamine levels and restored the time-of-day–dependent variations (Fig. 1B). These findings suggest that mast cells play a major role in the maintenance and temporal regulation of plasma histamine levels in mice.

Next, we investigated whether the mast cell–intrinsic clock was involved in circadian regulation of plasma histamine levels. In contrast to wild-type mice, mice harboring a loss-of-function mutation in *Clock (Clock*^*Δ19*/*Δ19*^ mice)^17^ did not exhibit circadian variations in plasma histamine levels, although their mean plasma histamine levels were similar to those of wild-type mice (Fig. 1A). In contrast to the results of reconstitution with wild-type BMMCs, mast cell–deficient W/Wv mice reconstituted with BMMCs derived from *Clock*^*Δ19*/*Δ19*^ mice failed to restore the circadian rhythm of plasma histamine levels (Fig. 1B). These results suggest that the mast cell–intrinsic clock mediates temporal regulation, but not maintenance, of plasma histamine levels at steady state in mice.

### Mast cells spontaneously release histamine according to a circadian rhythm *in vitro*, dependent on functional CLOCK

In rodent and human mast cells *in vitro*, a substantial proportion of newly formed histamine is spontaneously released into the extracellular environment, independently of degranulation^18^,^19^. To obtain mechanistic insight into the *in vivo* findings, we examined the kinetics of histamine release from mast cells *in vitro* using BMMCs derived from wild-type and *Clock*^*Δ19*/*Δ19*^ mice.

The circadian clocks of cultured cells can be synchronized by several procedures that activate various signaling pathways, including a simple media change or serum shock. Thereafter, the cells exhibit circadian cycles of gene expression for several consecutive days^11^,^12^,^13^. Hence, at least some cellular functions of circadian clocks can be investigated *in vitro*.

We showed previously that in *in vitro* culture, the mast cell clockwork is functional within a narrow window of time (0–48 hours after a media change for synchronization)^20^,^21^. This result was obtained by monitoring the bioluminescence of BMMCs derived from *Per2*^*Luc*^ knock-in mice^22^, which express a PER2–luciferase fusion protein (PER2^LUC^ BMMCs) (Supplementary Fig. S1). Therefore, we compared the kinetics of histamine release in PER2^LUC^ BMMCs *in vitro* within 0–48 hours after a media change in the presence of functional clock activity. Specifically, we measured histamine concentrations in the culture supernatants of wild-type and *Clock*-mutated PER2^LUC^ BMMCs cultured for 4, 8, 12, 16, 20, or 24 hours following the media change. Because histamine is degraded by fetal calf serum (Supplementary Fig. S2), we used serum-free medium for these *in vitro* experiments; under these conditions, histamine concentrations were stable for more than 24 hours (Supplementary Fig. S2). In addition, serum-free medium did not affect the mast cell clockwork (Supplementary Fig. S1A). We calculated the changes in histamine concentration in culture supernatants during the indicated intervals (4–8, 8–12, 12–16, 16–20, and 20–24 hours) and expressed each change as net histamine release from mast cells during that period of time.

Histamine release exhibited circadian variation in wild-type, but not *Clock*-mutated, BMMCs over 24 hours following a media change for synchronization, and this effect was enhanced by treatment of BMMCs with serum shock for 2 hours immediately before a media change (Supplementary Fig. S1, Fig. 1C). We also examined histamine release from fetal skin–derived connective tissue–type mast cells (FSMCs)^23^, a mature type of mast cells. We observed more abundant and clearer circadian variations in histamine release in these mature cells than in BMMCs (Fig. 1D).

Next, we confirmed that cromolyn sodium, an inhibitor of mast cell degranulation, did not affect histamine release from wild-type BMMCs and the viability of the cells (Supplementary Fig. S3A and B). Moreover, BMMCs did not express CD63, a marker of mast cell activation (degranulation), during the interval 0–24 hours after the media change (Supplementary Fig. S4). These results suggest that the mast cell clock is involved in the circadian release of histamine from mast cells *in vitro,* independently of degranulation.

### CLOCK temporally controls OCT3 expression in mast cells

To determine how the mast cell clockwork temporally regulates histamine release, we examined the mRNA and protein kinetics of the bidirectional organic cation transporter 3(OCT3)^24^ in mast cells. OCT3 functions as a transporter of histamine^25^ and implicated in the transportation (i.e., spontaneous release and/or uptake) of cytosol histamine in mast cells^26^ and basophils^27^.

**Figure 2:**
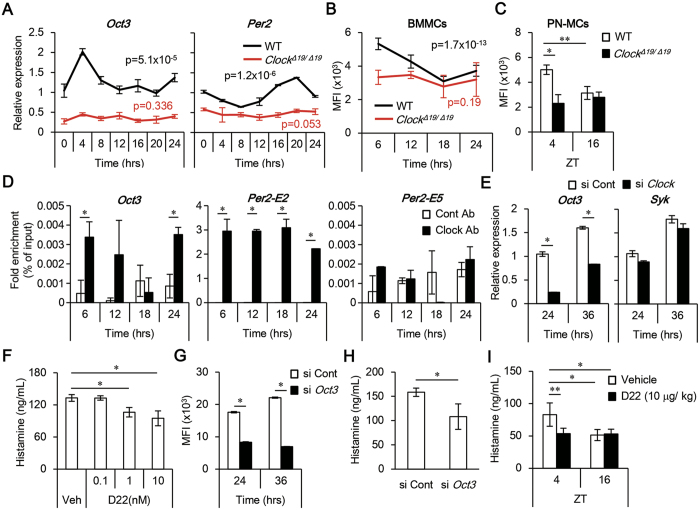
The mast cell clock regulates OCT3 expression, thereby temporally modulating plasma histamine levels. (**A)** Kinetics of *OCT3* and *Per2* mRNA expression levels in wild-type (WT) or *Clock*^*Δ19*/*Δ19*^ BMMCs. (n = 4) (One-way ANOVA). (**B)** Kinetics of OCT3 protein expression levels in wild-type or *Clock*^*Δ19*/*Δ19*^ BMMCs. (n = 10–12) (One-way ANOVA). (**C)** OCT3 protein expression levels in peritoneal mast cells isolated from wild-type or *Clock*^*Δ19*/*Δ19*^ mice at ZT4 or ZT16. (n = 5) (One-way ANOVA with Bonferroni’s test). (**D)** Detection of CLOCK binding to the promoter region of mouse *OCT3* (left panel) or E2 or E5 enhancer of mouse *Per2* (right panel) in wild-type BMMCs cultured for 6, 12 18, or 24 hours after a media change for synchronization. (n = 4) (Unpaired student’s t-test). (**E)** *OCT3* and *Syk* mRNA expression levels in wild-type BMMCs 24 and 36 hours after transfection with *Clock* siRNA. (n = 4) (Unpaired student’s t-test). (**F)** Histamine concentration in culture supernatants of wild-type BMMCs 24 hours after treatment with vehicle or D22. (n = 4) (One-way ANOVA with Bonferroni’s test). (**G)** OCT3 protein expression levels in wild-type BMMCs 24 and 36 hours after transfection with *OCT3* or control siRNA. (n = 4) (Unpaired student’s t-test). (**H)** Histamine concentration in culture supernatants of wild-type BMMCs 24 hours after *OCT3* or control siRNA transfection. (n = 6) (Unpaired student’s t-test). (**I)** Plasma histamine levels in mice treated with D22. Wild-type mice were i.p. injected with vehicle or D22 at ZT4, and then plasma histamine concentrations were measured by ELISA 24 hours (ZT4) or 36 hours (ZT16) after the treatment. (n = 5–6) (One-way ANOVA with Bonferroni’s test) Similar results (**A–I**) were obtained at least from 2 independent experiments. The values represent means ± SD, *p < 0.05, **p < 0.01.

*OCT3* mRNA was expressed according to a circadian rhythm in wild-type, but not *Clock*-mutated, BMMCs following a media change for synchronization (Fig. 2A). Likewise, the mRNA of a core circadian gene *Period2 (Per2*) exhibited circadian oscillations in wild-type, but not in *Clock*-mutated, BMMCs, in opposite phase to OCT3 (Fig. 2A). OCT3 protein levels also exhibited circadian oscillations in wild-type, but not *Clock*-mutated, BMMCs and wild-type FSMCs following a media change for synchronization (Fig. 2B, Supplementary Figs S5 and S6). Similarly, OCT3 protein expression in peritoneal mast cells isolated from wild-type, but not *Clock*-mutated, mice exhibited a time-of-day–dependent variation (Fig. 2C). We were unable to detect *OCT1* and *OCT2* mRNA expression in wild-type BMMCs (Supplementary Fig. S7). Thus, OCT3 expression exhibits temporal variations in mast cells both *in vitro* and *in vivo*, dependent upon CLOCK activity.

Several E-box–like elements to which the CLOCK/BMAL1 complex can bind^28^ are present in the promoter regions of mouse *OCT3* (Supplementary Fig. S8). ChIP assays revealed that CLOCK bound to the promoter of *OCT3* in the 6-hour and 24-hour, but not 12-hour and 18-hour, cultured (following a media change for synchronization) wild-type BMMCs (Fig. 2D). The integrity of ChIP samples was confirmed by monitoring constitutive binding of CLOCK to the promoter of the *Per2* gene containing the non-canonical E-box enhancer 2 (E2) sequence, but not the E5 sequence, in wild-type BMMCs, as previously reported^29^ (Fig. 2D). Consistent with this, *Clock* siRNA, but not control siRNA, significantly decreased *OCT3* mRNA levels in wild-type BMMCs (Supplementary Fig. S9 and Fig. 2E), but had no effect on *Syk* mRNA.

To determine the functional roles of OCT3 in histamine release from mast cells, we inhibited OCT3 in BMMCs using the OCT inhibitor Decynium-22 (D22)^27^. Histamine release from D22-treated wild-type BMMCs decreased in a dose-dependent manner relative to release from vehicle-treated BMMCs (Fig. 2F). D22 did not affect cell the viability of BMMCs at the concentrations used (Supplementary Fig. S10). Knockdown of *OCT3* using siRNA significantly decreased OCT3 protein levels and histamine release from wild-type BMMCs (Supplementary Fig. S11, Fig. 2G and H). *In vivo*, plasma histamine levels significantly decreased in mice treated with D22 relative to those in mice treated with vehicle (Fig. 2I). Collectively, these results suggest that CLOCK temporally controls OCT3 expression in mast cells, thereby regulating spontaneous histamine release from mast cells.

### Mice housed in an aberrant circadian light environment do not exhibit temporal variations in OCT3 expression or plasma histamine levels

If OCT3 expression in mast cells and plasma histamine levels is under the control of the circadian clock, functional disruption of the circadian clock system by housing mice under irregular circadian lighting environments^11^,^12^,^13^ should affect these outputs.

**Figure 3:**
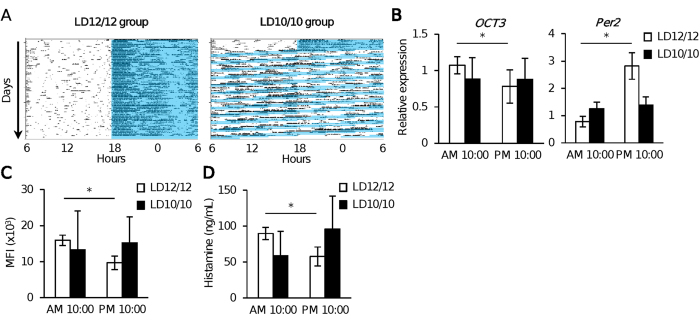
Aberrant light/dark condition affects OCT3 expression in mast cells and plasma histamine levels. (**A)** General locomotor activity of mice housed under a 10-hour light/10-hour dark (LD10/10) cycle (right) or a normal LD12/12 cycle (left). Locomotor activity was recorded as indicated by black dots over a 60-day period. Representative single-plotted actograms of mice are shown. In mice housed under a LD10/10 cycle, mice were kept under a consistent LD12/12 cycle in the first 7 days, and then were exposed to LD10/10 cycles for 60 days, as indicated by the black arrow on the right. Dark phase is indicated by blue shadow on each record. Please note the clear day-night differences in the locomoter activity of the mice kept under LD12/12 cycle (mice are nocturnal animals), but the activity became arrhythmic (random) in mice kept under LD10/10 cycle. (**B)** *OCT3* and *Per2* mRNA expression at ZT4 and ZT16 in the skin of mice housed under LD10/10 and LD12/12 cycles. (n = 6–8). (**C)** OCT3 protein expression at ZT4 and ZT16 in the peritoneal mast cells isolated from mice housed under LD10/10 and LD12/12 cycles. (n = 8–10). (**D)** Plasma histamine levels at ZT4 and ZT16 in mice housed under LD10/10 and LD12/12 cycles. (n = 8–10). The values represent means ± SD. *p < 0.05 (Unpaired student’s t-test) Similar results (**A–D**) were obtained at least from 2 independent experiments.

Mice housed under a 10-hour light/10-hour dark (LD10/10) cycle for 8 weeks, but not mice housed under a normal LD12/12 cycle, became arrhythmic or free-running in locomotor activity^30^ (Fig. 3A and Supplementary Fig. S12). For the following experiments, we selected mice that became arrhythmic. *OCT3* mRNA expression in skin and protein expression in peritoneal mast cells varied with the time of day in mice housed under a LD12/12 cycle, but this pattern was absent in mice housed under a LD10/10 cycle for 8 weeks (Fig. 3B and C). The time-of-day–dependent variation of plasma histamine levels were also absent in mice housed under a LD10/10 cycle, but not a LD12/12 cycle, for 8 weeks (Fig. 3D). These results support the notion that OCT3 expression in mast cells and plasma histamine levels is under the control of the circadian clock *in vivo*.

### Mice suffering from restraint stress exhibit de-synchronization of the mast cell clockwork, concomitant with the absence of temporal variations in OCT3 expression and plasma histamine levels

**Figure 4:**
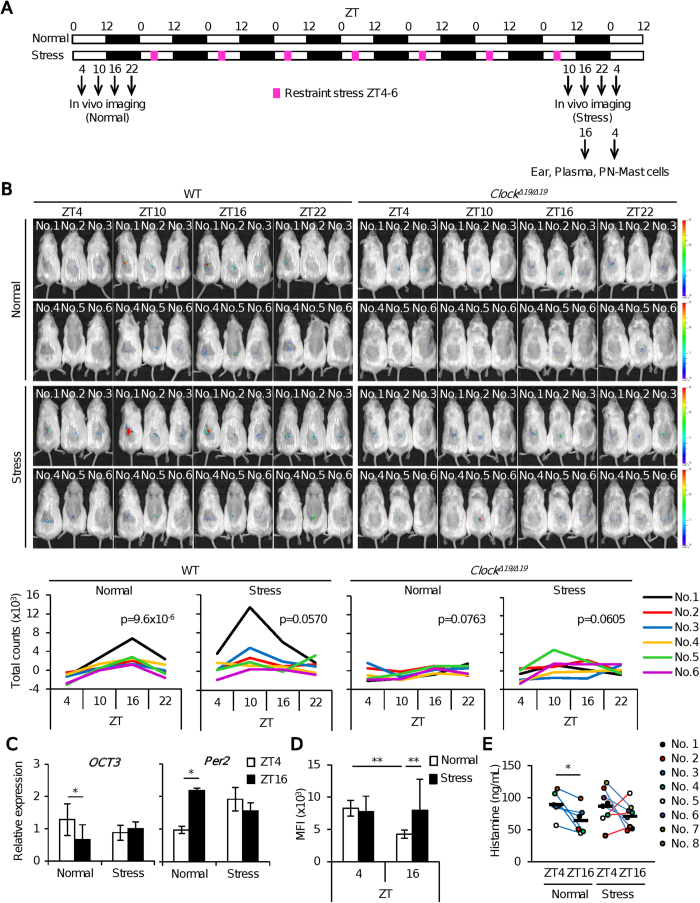
Sub-acute restraint stress results in de-synchronization of the mast cell clockwork, concomitant with the absence of temporal variations in OCT3 expression and plasma histamine levels. (**A**) Experimental protocol. (**B**) Top: Representative pictures of *in vivo* imaging of mast cell–deficient mice reconstituted with subcutaneous injections of BMMCs derived from PER2^LUC^ knock-in mice, with or without *Clock* mutation, at the indicated time points. Bottom: A quantitative analysis of the data. (n = 6) (One-way repeated measures ANOVA). (**C**) *OCT3* and *Per2* mRNA expression at ZT4 and ZT16 in the skin of control and stressed mice. (n = 8–10) (Unpaired student’s t-test). (**D**) OCT3 protein expression in the peritoneal mast cells isolated from control and stressed mice at ZT4 and ZT16. (n = 8–10) (One-way ANOVA with Bonferroni’s test). (**E**) Plasma histamine levels at ZT4 and ZT16 in individual control and stressed mice. (n = 6–8) (Paired student’s t-test) The values represent means ± SD, *p < 0.05, **p < 0.01. Similar results (**B**–**E**) were obtained at least from 2 independent experiments.

Previously, using *in vivo* imaging analysis, we showed that mice exposed to restraint stress for 2 hours at the beginning of the light period (ZT4–6) every day for 7 days exhibited modulations of peripheral clock activity in the liver and kidney in an organ-specific manner^31^. Therefore, we investigated whether sub-acute restraint stress would also modulate the mast cell clockwork and affect daily variations in OCT3 expression in mast cells and plasma histamine levels (Fig. 4A).

*In vivo* imaging using mast cell–deficient W/Wv mice subcutaneously reconstituted with PER2^LUC^ BMMCs revealed that PER2^LUC^ bioluminescence exhibited a time-of-day–dependent variation, with a maximum at ZT16 and a minimum at ZT4 (Fig. 4B), demonstrating that the functional mast cell clockwork can be visualized *in vivo*^20^,^21^. Consistent with this, the mast cell–deficient W/Wv mice subcutaneously reconstituted with PER2^LUC^ BMMCs harboring a *Clock*-mutation did not exhibit the daily variations (Fig. 4B). Furthermore, we found that mast cell–deficient W/Wv mice subcutaneously reconstituted with PER2^LUC^ BMMCs suffering from restraint stress lacked daily variations in PER2^LUC^ levels in mast cells (Fig. 4B). Similarly, mast cell–deficient W/Wv mice subcutaneously reconstituted with PER2^LUC^ BMMCs harboring the *Clock*-mutation also lacked the daily variations following restraint stress (Fig. 4B).

Consistent with earlier findings, daily variations in *OCT3* mRNA in the skin and protein expression in peritoneal mast cells were absent in wild-type mice suffering from restraint stress (Fig. 4C and D). Daily variations of plasma histamine levels were also absent in restrained wild-type mice (Fig. 4E). These findings suggest that sub-acute physical stress causes de-synchronization of the mast cell clockworks, resulting in the absence of daily variations in OCT3 expression and plasma histamine levels, and also support the notion that these outputs are under the control of the circadian clock.

### Plasma histamine levels are correlated with the degree of mast cell–mediated allergic reaction

**Figure 5:**
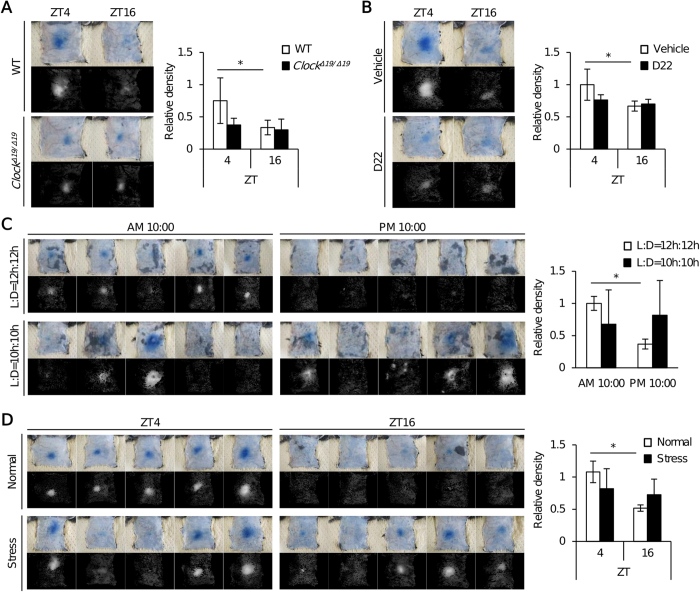
Plasma histamine levels are correlated with the degree of compound 48/80–induced plasma extravasation. (**A)** Compound 48/80–induced plasma extravasation at ZT4 and ZT16 in wild-type and *Clock*^*Δ19*/*Δ19*^ mice. (n = 6). (**B)** Compound 48/80–induced plasma extravasation at ZT4 and ZT16 in vehicle- or D22-treated wild-type mice as described in Fig. 2. (n = 5). (**C**) Compound 48/80–induced plasma extravasation at AM 10:00 and PM 10:00 in mice housed under LD10/10 and LD12/12 cycles as described in Fig. 3. (n = 5–8). (**D)** Compound 48/80–induced plasma extravasation at ZT4 and ZT16 in control and sub-acute restraint-stressed mice as described in Fig. 4. (n = 5–10) Representative pictures and quantitative analysis of the data are shown. The values represent means ± SD. *p < 0.05 (Unpaired student’s t-test) Similar results (**A–D**) were obtained at least from 2 independent experiments.

Histamine is an important mediator of the initiation of allergic reactions^32^, and plasma histamine levels are correlated with the severity of allergic symptoms^10^,^33^. Therefore, we investigated whether temporal variations of plasma histamine levels were correlated with the degree of compound 48/80–induced mast cell–dependent plasma extravasation. For this purpose, we compared the extents of compound 48/80–induced mast cell–dependent plasma extravasation at ZT4 and ZT16 between wild-type and *Clock*-mutated mice, between D22-treated and vehicle-treated mice, between mice housed under LD12/12 and LD10/10 cycles, and between mice suffering from restraint stress and unrestrained controls. Overall, we found that the degree of compound 48/80–induced plasma extravasation was correlated with basal plasma histamine levels (Fig. 5A–D).

To exclude the possibility that mast cell responses to compound 48/80 exhibit temporal variations, thereby generating a time-of-day–dependent variation in compound 48/80–induced plasma extravasation *in vivo*, we examined compound 48/80–induced degranulation in wild-type BMMCs cultured for 12 or 24 hours following a media change for synchronization. The levels of compound 48/80–induced β-hexosaminidase release were comparable between wild-type BMMCs cultured for 12 or 24 hours, and were unaffected by *Clock* mutation (Supplementary Fig. S13). In addition, there was little circadian oscillation in the expression of Mrgprb2, the receptor for compound 48/80^34^, in wild-type BMMCs and in the skin of wild-type mice (Supplementary Fig. S14A and B). Consistently, *Clock* knockdown with siRNA did not affect Mrgprb2 mRNA levels in wild-type BMMCs (Supplementary Fig. S14C). Furthermore, serum monocyte chemoattractant protein (MCP)-1 [CCL2] levels in wild-type mice treated with compound 48/80 were comparable between mice treat at ZT4 or ZT16 (Supplementary Fig. S15). Thus, mast cell responses to compound 48/80 do not appear to exhibit temporal variations. Collectively, these findings suggest that plasma histamine might potentiate compound 48/80–mediated plasma extravasation downstream of mast cell activation by this reagent.

## Discussion

The results of this study demonstrate that mast cells play a major role in maintaining base-line plasma histamine levels, and that the circadian clock in mast cells contributes to temporal regulation of plasma histamine levels at steady state in mice, at least in part by controlling OCT3 expression (Figs 1 and 2). In support of this notion, environmental factors that de-synchronize the mast cell clockwork, such as aberrant light/dark conditions or sub-acute restraint stress, affected OCT3 expression and plasma histamine levels (Figs 3 and 4). Additionally, baseline plasma histamine levels were correlated with the degree of compound 48/80–induced plasma extravasation (Fig. 5). Therefore, we propose that plasma histamine levels may potentiate mast cell–mediated allergic reaction.

Our findings indicate that mast cells are the main cell type responsible for maintaining plasma histamine levels in mice at steady state (Fig. 1). This notion may be supported by a number of clinical reports showing that patients with mastocytosis have higher plasma histamine levels than normal subjects and exhibit a diurnal variation in plasma histamine levels^35^,^36^.

Because histamine in plasma is rapidly metabolized (~5 minutes)^37^, we assume that plasma histamine levels at steady state largely reflects constitutive release of histamine from tissue mast cells into the circulation.

Histamine is synthesized in the cytosol of mast cells by histidine carboxylase, and is transported into granules by vesicular amine tranporter-2 (VMAT2); however, a substantial portion (or excess amounts) of histamine is spontaneously released to extracellular environment^38^. OCT3 is a transporter for organic cations with low affinity but high capacity^24^,^25^, and is implicated in the transport of cytosol histamine in mast cells^26^ and basophils^27^. Hence, we investigated the link between CLOCK and OCT3 expression/function in mast cells (Fig. 2). The results revealed that *OCT3* is a *Clock*-controlled gene that temporally regulates both spontaneous histamine release from mast cells and plasma histamine levels in mice. Consistent with the tissue-selective expression of OCT1 and OCT2^24^, we did not detect mRNA expression of these genes in wild-type BMMCs (Supplementary Fig. S7), suggesting the predominant role of OCT3 in histamine release from mast cells.

Stressful conditions predispose humans to allergic reactions^39^,^40^,^41^. Given that baseline plasma histamine levels were correlated with the degrees of mast cell–mediated allergic reactions (Fig. 5), we speculate that stressful conditions might influence the degree of mast cell–mediated allergic reaction, at least in part by affecting the mast cell clock–dependent regulation of plasma histamine levels. It will be thus interesting to investigate whether not only sub-acute restraint stress but also chronic stress (e.g. a range of social and psychosocial stress) can critically modulate the mast cell clock–dependent regulation of plasma histamine levels and affect temporal profiles or intensity of mast cell–mediated allergic reactions. In addition, the findings that stress can affect plasma histamine levels might underlie the wide inter-individual range or loss of circadian variations in plasma histamine levels in normal subjects or patients with asthma in some reports^8^,^42^.

The degrees of compound 48/80–induced plasma extravasation were correlated with plasma histamine levels (Fig. 5). Given that compound 48/80–induced mast cell activation (β-hexaminitase release and MCP-1 [CCL2] release) and expression of the compound 48/80 receptor (Mrgprb2) in mast cells did not exhibit temporal variations (Supplementary Figs S13–15), we assume that plasma histamine potentiates compound 48/80–induced plasma extravasation downstream of mast cell activation by this drug. For instance, baseline plasma histamine might potentiate endothelial cell responses to compound 48/80–induced histamine or other chemical mediators from mast cells via mechanisms yet to be elucidated. Potentiation (sensitization) of cellular responses by histamine has been reported in patients with irritable bowel syndrome, in which pre-exposure of sensory neurons from the colon to histamine increases TRPV4-mediated intracellular signaling, thereby increasing visceral hypersensitivity^43^. However, the direct causal relationship between plasma histamine and the degree of allergic reactions remains to be determined.

We showed previously that the extent of IgE-mediated degranulation in mast cells exhibits temporal variations with peaks in the resting phase, dependent upon circadian regulation of FcεRI expression and signaling by mast cell clock activity^21^,^44^. These findings suggest that the mast cell clockwork temporally gates mast cell responses to IgE, thereby causing the nighttime exacerbation of IgE-/mast cell–mediated allergic symptoms^44^. This study suggests that circadian regulation of plasma histamine levels by the mast cell clockwork may also be associated with temporal or stress-induced variations in allergic reactions. Hence, the current findings provide another layer of complexity underlying the temporal regulation of allergic reaction.

In summary, the mast cell–intrinsic clock plays a predominant role in circadian regulation of steady-state plasma histamine levels in mice, which can be modulated by stress. Additionally, this mechanism may be associated with the nighttime- or stress-induced variations of allergic symptoms. To the best of our knowledge, this is the first study to reveals a regulatory mechanism of plasma histamine levels at steady state and its possible roles in the pathologies of allergy.

## Methods

### Mice

Male 5- to 6-week-old C57BL/6 mice (Japan SLC, Tokyo, Japan), mast cell-deficient WBB6F1-W/Wv mice (Japan SLC, Tokyo, Japan), *Per2*^*Luciferase*^ (*Per2*^*Luc*^) knock-in mice^22^ (C57BL/6 background) which express PERIOD2 (PER2) as a luciferase fusion protein, and C57BL/6 *Clock*^*Δ19*/*Δ19*^ mice^17^ were used. *Clock*^*Δ19*/*Δ19*^ mice were mated to generate *Clock*^*Δ19*/*Δ19*^ *Per2*^*Luc*^ knock-in mice. *Clock*^*Δ19*/*Δ19*^ mice have an A to T point mutation in the 5′ splice site of intron 19 and, as a consequence, an in-frame deletion of the entire exon 19 (*Clock*^*Δ19*/*Δ19*^), which results in the loss of normal transcriptional activity^17^. All animal experiments were approved by the Institutional Review Board of the University of Yamanashi and carried out according to the guidelines.

### Reagents

Chromolyn sodium and Decynium 22 (D22) were purchased from Merck Millipore (Darmstadt, Germany). Histamine was purchased form Sigma-Aldrich (St. Louis, MO).

### Preparation of bone marrow-derived mast cells (BMMCs) and fetal skin-derived mast cells (FSMCs)

Bone marrow-derived mast cells (BMMCs) were generated from the femoral bone marrow cells of male mice as previously described^45^. Fetal skin-derived mast cells (FSMCs) were generated as previously described^23^.

### FACS staining

BMMCs or peritoneal mast cells were incubated for 15 minutes with rat-anti-mouse Abs to CD16/32 (2.4 G; BD Biosciences, San Diego, CA) to block nonspecific binding, and then were stained with FITC-conjugated anti-mouse FcεRIα (MAR-1; eBioscience, San Diego, CA) and a PE-conjugated anti-mouse c-Kit Ab (2B8; BD PharMingen, San Diego, CA) in PBS for 30 minutes on ice. For some experiments, APC-conjugated anti-mouse CD63 Ab (HMa; eBioscience, San Diego, CA) was used for staining. After being washed with PBS, the stained cells (live-gated on the basis of forward and side scatter profiles) were analyzed on a quantitated by BD Accuri™ C6 flow cytometry (Becton Dickinson) and data were processed using the BD Accuri™ CFlow software program (Becton Dickinson).

### Subcutaneous reconstitution of mast cells

Mast cell-deficient WBB6F1-W/Wv mice (Japan SLC, Tokyo, Japan) were reconstituted with subcutaneous injections of BMMCs (1.5 × 10^6^/mouse) derived from *Per2*^*Luc*^ mice with or without *Clock*^*Δ19*/*Δ19*^ mutation (PER2^LUC^ BMMCs or *Clock*^*Δ19*/*Δ19*^ PER2^LUC^ BMMCs). Six weeks after reconstitution, the mice were used for experiments.

### Measurement of histamine levels

The amounts of histamine in the mouse plasma and in the culture supernatants were measured with histamine EIA kit (Oxford Biomedical research, Inc., Oxford, MI, USA).

For some experiments, the changes of histamine concentrations in the culture supernatants during the indicated culture duration (e.g. 4~8 hours, 8~12 hours, 12~16 hours, 16~20 hours, 20~24 hours) were calculated and expressed each of them as the amounts of net histamine release from mast cells during the culture duration. The plasma or culture supernatant samples were not taken from the same mice or cultures, but from the different mice or cultures except the experiments in Fig. 4E.

### Detection of OCT3 in mast cells (Intracellular FACS staining)

BMMCs, FSMCs or peritoneal mast cells were collected and resuspended in 0.2 ml Fixation and permeabilization buffer (eBioscience, San Diego, CA). The cells were fixed for 30 minutes at 4 °C. The permeabilized cells (1 × 10^6^) were mixed with 100 μl permeabilization buffer (eBioscience, San Diego, CA) containing 1 μg/ml anti-mouse SLC22A3 (OCT3) antibody (Abcam, Cambridge, MA) or isotype rabbit IgG (Cell Signaling Technology, Inc.) and incubated for 30 minutes at 4 °C. The cells were then washed with permeabilization buffer followed by incubation with Alexa Fluor^®^ 647-conjugated Goat anti-rabbit IgG antibody (2 μg/ml, Life science) for 30 minutes at 4 °C. The cells were washed with permeabilization buffer and then resuspended in 0.3 ml permeabilization, and evaluated by FACS analysis.

### Measurement of bioluminescence in BMMCs generated from *Per2* ^*Luc*^ mice

BMMCs generated from *Per2*^*Luc*^ knock-in mice were placed in a 35-mm Petri dish following centrifugation at 1500 rpm for 5 minutes, and were incubated at 37 °C. The bioluminescence was monitored at 10 minutes intervals for 120 hours using a dish-type luminometer (Kronos DioAB-2550, ATTO Inc., Tokyo, Japan) as previously described^20^,^21^.

### Quantitative real-time PCR (Q-PCR)

A quantitative real-time PCR analysis using cDNA from BMMCs was performed using the StepOne™ real-time PCR system (Applied Biosystems, Foster City, CA, USA) according to the manufacturer’s instructions, using primers and probes for mouse *OCT1, OCT2, OCT3, Clock, Period2 (Per2), Syk, Mrgprb2,* and *GAPDH* (Applied Biosystems, Foster City, CA) as previously described^20^. The ratio of the indicated genes to that of *GAPDH* was calculated, and the relative expression levels are shown.

### Peritoneal mast cell assay

Peritoneal mast cells were collected from wild-type mice or some experimental type mice models and the OCT3 levels on mast cells gated by FcεRIα and c-kit were immediately assessed by FACS analysis.

### Chromatin immunoprecipitation (ChIP) assay

ChIP assay was performed as previously described^21^. In brief, nuclear fractions were extracted as chromatin from sonicated cells. After preclearance with protein G agarose/salmon sperm DNA (Millipore, Billerica, MA), chromatin fractions were incubated with anti-mouse/human KAT13D/CLOCK antibody ChIP grade (rabbit polyclonal IgG) (ab3517, Abcam, Cambridge, MA), anti-mouse SLC22A3 (OCT3) Ab (ab191446, Abcam, Cambridge, MA) or control purified rabbit IgG (no. 026102, Invitrogen, Inc., Carlsbad, CA). The “input” samples were DNA extracts prepared from untreated chromatin, and DNA was extracted from immunoprecipitated chromatin. Equivalent masses of immunoprecipitated and input DNA were analyzed by real-time PCR using primers and a TaqMan probe for the promoter region of *OCT3* (Supplementary Fig. S7), *mPer2-E2*, and *mPer2-E5* (ref. 22) as follows:

*OCT3*

Sense (5′-GGAGCTGGAGGAATGTGATGAC-3′),

Antisense (5′-CTCCAGGAACAGAGTTTCATACCT-3′),

TaqMan probe (5′-FAM-CTGCACCTGCACCTGC-MGB-3′).

*mPer2-E2*,

Sense (5′-CCACCAATTGATGAGCGTAGC-3′),

Antisense (5′-CGTCGCCCTCCGCTG-3′),

TaqMan probe (5′-FAM-TCACGTTTTCCACTATGTG-MGB-3′).

*mPer2-E5*,

Sense (5′-TCCTGCCACATTGAGATTTGG-3′),

Antisense (5′-GTGATTGCCCCACACTCACA-3′),

TaqMan probe (5′-AAGAGATGGCACGTTAGT-MGB-3′).

Data are presented as the ratio of the cycling threshold value of immunoprecipitated DNA to that of input DNA.

### siRNAs

All siRNAs were purchased from Invitrogen, Inc. (Carlsbad, CA). The negative control (Stealth^TM^ RNAi; Negative control Low GC Duplex #2) consists of a 20–25 nucleotides scrambled sequence, which does not target any known cellular mRNA. Specific siRNAs against *Clock* (Stealth^TM^ RNAi; ClockMS203030 3_RNAI) or *OCT3* (Stealth^TM^ RNAi; Slc22a3MSS209120 3_RNAI) was purchased as a pool of three 25 nucleotides target specific siRNAs.

### Transfection by electroporation

Wild-type BMMCs were plated at 1 × 10^6^ cells/ml in 10-cm Petri dish, 24 hours before the transfection. Transfection of BMMCs were performed with Mouse Macrophage Nucleofector kit^®^ (VPA-1009; Amaxa Biosystems, now Lonza, Basel, Switzerland). Cells were suspended in Nucleofector^®^ Solution to a final concentration of 2 × 10^6^ cells/100 μl. The cells were transfected using 500 nM of the negative control or specific siRNAs. In Amaxa certified cuvettes, the cells were nucleofected by using the program Y-001. The nucleofected cells were transferred to 1900 μl of pre-warmed medium in 12-well plates and incubated for 24 hours.

### Locomoter activity

General locomoter activity of the mice was recorded with an infrared radiation sensor (F5B, Omron, Tokyo, Japan) and analyzed with CLOCKLAB software (Actimetrics, Wilmette, IL, USA) as described previously^46^.

### Application of restraint stress

PER2^LUC^ BMMCs or *Clock*^*Δ19*/*Δ19*^ PER2^LUC^ BMMCs reconstituted mast cell-deficient WBB6F1-W/Wv or wild-type mice were subjected to restraint stress using a wire-mesh bag (3 × 6 × 12 cm) clipped to their home cage as previously described^31^. Mice were restrained during the resting period, ZT4–6 (lights were turned on at ZT0), for 7 consecutive days.

### *In vivo* imaging

Mast cell-deficient WBB6F1-W/Wv mice (Japan SLC, Tokyo, Japan) were reconstituted with subcutaneous injections of BMMCs (1.5 × 10^6^/mouse) derived from *Per2*^*Luc*^ mice with or without *Clock*^*Δ19*/*Δ19*^ mutation (PER2^LUC^ BMMCs or *Clock*^*Δ19*/*Δ19*^ PER2^LUC^ BMMCs). Six weeks after reconstitution, 20 μl of luciferin (50 mg/ml) was subcutaneously administered at the indicated time points, and then the bioluminescence emission from the mice was recorded as described previously^20^,^21^.

### Induction of compound 48/80-mediatd plasma extravasation

Following the i.v. injection of 0.5% Evans blue dye, the mice were subcutaneously injected with compound 48/80 (1 μg/20 μl) (Sigma-Aldrich, St. Louis, MO). Vascular permeability was visualized 30 minutes later by the blue staining of the injection sites on the reverse side of the skin. These staining sites were digitalized using a high-resolution color camera (digital cameraIXY3, Canon Inc., Tokyo, Japan) and the images were saved in Windows photo viewer as 8-bit color-scale JPEG files. The open source ImageJ 1.43 software package (NIH, USA) was used for the image analysis, as described previously^20^,^21^. Briefly, color-scale images exported from Windows photo viewer were converted to HSB (“hue/saturation/brightness”) stack type images using the Image tool. Thereafter, the HSB stack images were split into hue, saturation, and brightness images, respectively. Only blue color stained-areas were selected from the hue image using the threshold tool. These images were then combined with the saturation image and the density values for the blue color stained-areas, and were measured using the analyze tool.

### Compound 48/80-induced β-hexosaminidase release assay

BMMCs (1 × 10^6^ cells/ml) were stimulated for 40 minutes at 37 °C with 10 μg/ml compound 48/80 (Sigma-Aldrich, St. Louis, MO). The β-hexosaminidase release assay was performed and analyzed as described previously^18^.

### Serum monocyte chemoattractant protein (MCP)-1 levels

Serum samples were collected from the mice subjected to the development of PCA reactions (30 minutes after the antigen challenge). The amounts of MCP-1 (CCL2) in the serum were measured using a mouse MCP-1 ELISA kit (R&D, Minneapolis, MN).

### Statistical analysis

The statistical analyses were performed using the paired or the unpaired Student’s *t-*test for two group-comparisons. For multigroup comparisons, we applied one-way repeated measures ANOVA or one-way ANOVA with post hoc testing using Bonferroni’s multiple comparison test. A value of p < 0.05 was considered to be significant unless otherwise indicated.

## Additional Information

**How to cite this article**: Nakamura, Y. *et al*. Regulation of plasma histamine levels by the mast cell clock and its modulation by stress. *Sci. Rep.* **7**, 39934; doi: 10.1038/srep39934 (2017).

**Publisher's note:** Springer Nature remains neutral with regard to jurisdictional claims in published maps and institutional affiliations.

## Supplementary Material

Supplementary Figures
