# Supplementary material for: Regulation of plasma histamine levels by the mast cell clock and its modulation by stress
Source: Sci Rep. 2017 Jan 11;7:39934. doi: 10.1038/srep39934 (PMC5225447; doi:10.1038/srep39934)
Supplement: Supplementary Figures [file srep39934-s1.pdf]

Supplementary information

Regulation of plasma histamine levels by the mast cell clock and its modulation by stress

Yuki Nakamura,[1] Kayoko Ishimaru,[1] Shigenobu Shibata,[2] Atsuhito Nakao[1,3, *]

[1]Department of Immunology, University of Yamanashi Faculty of Medicine, 1110 Shimokato, Chuo, Yamanashi 409-3898, Japan
[2]Department of Physiology and Pharmacology, School of Advanced Science and Engineering, Waseda University, 2-2, Wakamatsu-cho, Shinjuku-ku, Tokyo, 162-8480, Japan
[3]Atopy Research Center, Juntendo University School of Medicine, 2-1-1 Hongo, Bunkyo-ku, Tokyo, 113-8421, Japan

***Corresponding author:** Atsuhito Nakao,

Department of Immunology, Faculty of Medicine, University of Yamanashi, 1110 Shimokato, Chuo, Yamanashi 409-3898, Japan
TEL: +81-55-273-6752, FAX: +81-55-273-9542, E-mail: anakao@yamanashi.ac.jp

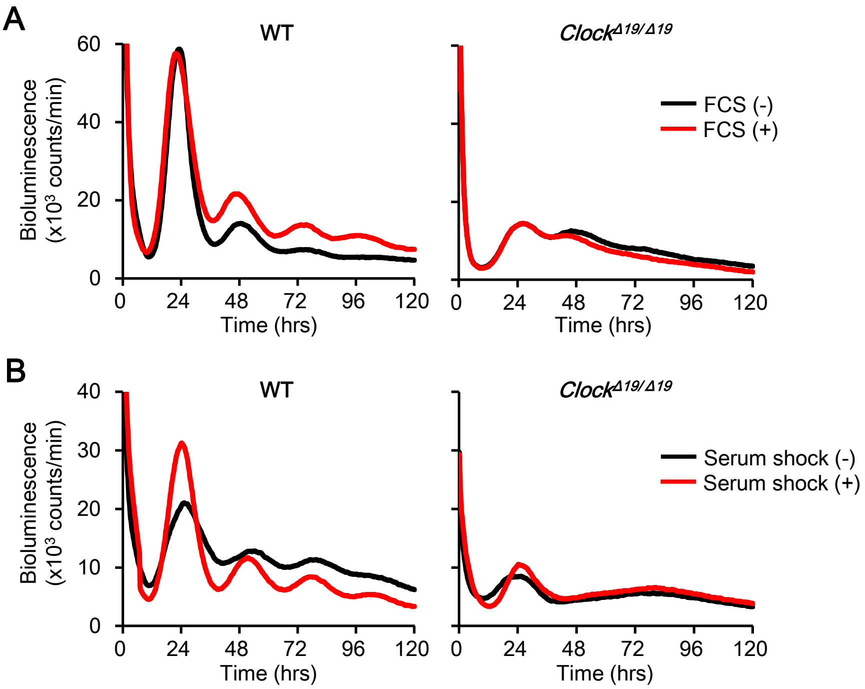

Supplementary Figure S1. **Mast cell clockwork is functional *in vitro* culture for 0–48 hours after a media change**

PER2^{LUC} bioluminescence of BMMCs derived from *Per2^{Luc}* knock-in mice (PER2^{LUC} BMMCs) was monitored for 120 hours after a media change for synchronization.

A. The PER2^{LUC} bioluminescence was monitored for 120 hours using PER2^{LUC} BMMCs cultured in medium with or without fetal calf serum (FCS).

B. The PER2^{LUC} bioluminescence was monitored for 120 hours using PER2^{LUC} BMMCs with or without serum shock treatment. The cells were stimulated with medium containing 50% horse serum for 2 hours before the media change and thereafter the bioluminescence was monitored for 120 hours. These data were previously published (13, 14), but are presented here to help readers follow the logic of this manuscript.

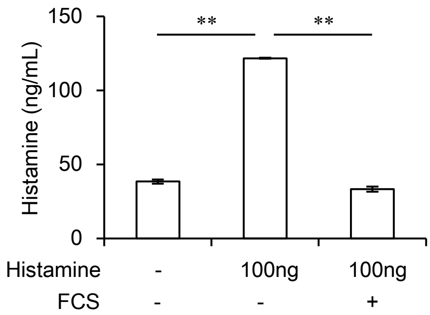

Supplementary Figure S2. **Histamine is stable in serum-free medium for more than 24 hours *in vitro***

Histamine was added to the serum-free medium and incubated at 37° C
for 24 hours with or without fetal calf serum (FCS). Then, histamine concentrations in the medium were measured by ELISA (n = 4). Please note that histamine concentrations did not significantly change for 24 hours after addition of histamine to medium without FCS.

One-way ANOVA with Bonferroni's test; Values represent means ± SD. *p < 0.05, **p < 0.01.
Similar results were obtained at least from 2 independent experiments.

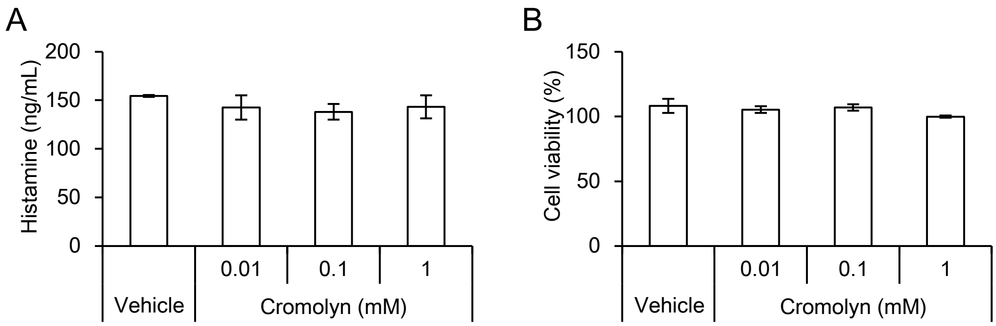

Supplementary Figure S3. **Cromolyn sodium, an inhibitor of mast cell degranulation, does not affect histamine release from wild-type BMMCs**

A. Wild-type BMMCs were cultured for 24 hours following a media change for synchronization in the presence or absence of cromolyn sodium. The histamine concentration in culture supernatants of wild-type BMMCs were then measured by ELISA. (n = 4)

B. The viability of wild-type BMMCs cultured for 24 hours following a media change for synchronization in the presence or absence of cromolyn sodium were determined by WST assay. (n = 4)

One-way ANOVA with Bonferroni's test; Values represent means ± SD. *p < 0.05, **p < 0.01. Similar results (**A**, **B**) were obtained at least from 2 independent experiments.

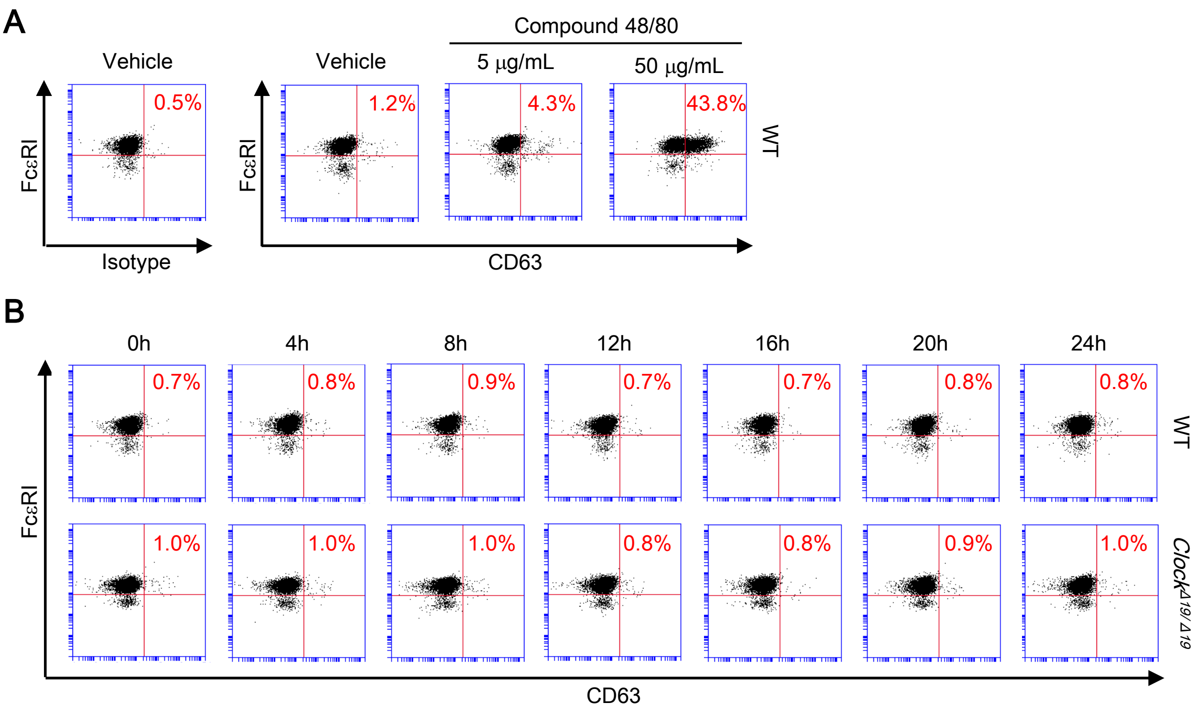

Supplementary Figure S4. **Cultured BMMCs do not express CD63, a marker of mast cell activation (degranulation), for 0–24 hours following a media change**

A. **B**. Wild-type or *Clock*$^{\Delta 19/\Delta 19}$ BMMCs were cultured for 24 hours following a media change for synchronization. At the indicated times, the frequencies of FcεRIα^{+}CD63^{+} cells were detected by flow cytometry (**B**). As a positive control, the cells were stimulated with compound 48/80 (**A**). (n = 6)

Similar results (**A**, **B**) were obtained at least from 2 independent experiments.

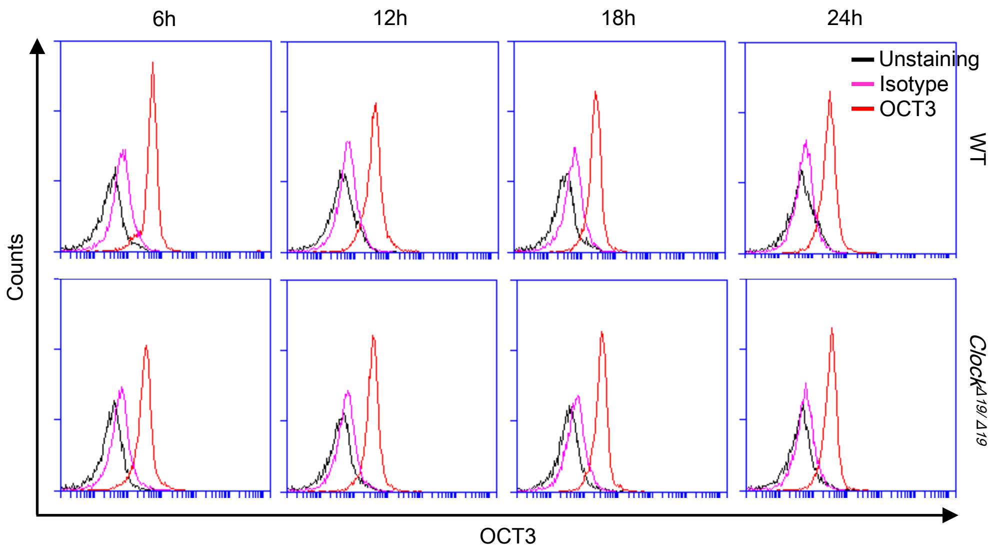

Supplementary Figure S5. OCT3 protein expression levels in wild-type or *Clock*$^{\Delta 19/\Delta 19}$ BMMCs
Wild-type BMMCs or *Clock*$^{\Delta 19/\Delta 19}$ BMMCs were stained by anti-OCT3 antibody or isotype rabbit IgG and analyzed by flow cytometry. OCT3 protein expression levels in BMMCs were specifically detected by anti-OCT3 antibody.
Similar results were obtained at least from 2 independent experiments.

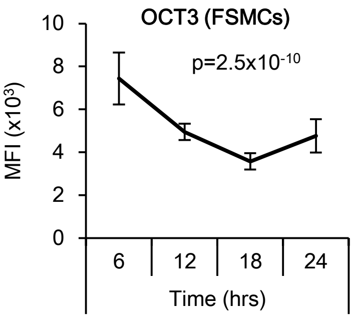

Supplementary Figure S6. **Kinetics of OCT3 protein expression levels in wild-type FSMCs**
Wild-type fetal skin-derived connective tissue-type mast cells (FSMCs) were cultured for 24 hours following a media change for synchronization. OCT3 protein expression levels were analyzed at the indicated time points by flow cytometry. (n = 9)
P values are shown for one-way repeated measures ANOVA; values represent means ± SD. Similar results were obtained at least from 2 independent experiments.

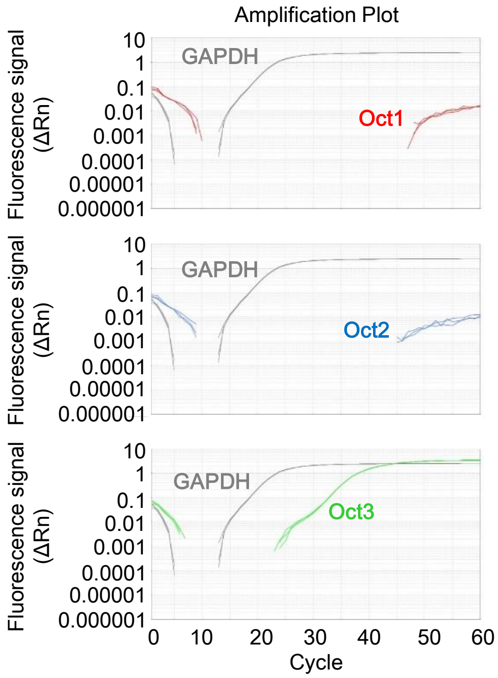

Supplementary Figure S7. **BMMCs do not express *OCT1* and *OCT2* mRNAs**

OCT1, *OCT2, and OCT3* mRNAs expressed in wild-type BMMCs were quantitated by qPCR. Representative qPCR data are shown. Similar results were obtained by 2 independent experiments.

gaggaggagg tgggctggag aggggagacc caggctgggg attcagcagg cagttacaaa caggaccaga ggggcgtgtg
agatatgcag attcagaaca cagtgagtga ggagtaggtg gtttgtgcgt gtacacgcat gcgtgtgtgt gtgtgtgggg
gggtgtcgga ggagaggtag agagagatgg ctgtcttaag ctgtatggtt ttggcagttc ttgggcccac ccatggttgc
tcagtggcaa agagcatctg tggtgacgat tattgaatga aaccctgact acttttctgg tactattcct cttgccaatc
catttagtct tcacagagac cctatgaagt aggtgtgtta ttagacttat ttaaaagatg gaaaaactgg ggctggccag
ttaggtttct cagcatcccg agtagtaagg gtgagagatg ctggagcaag gcagcctggc tctagcattc ttgctgtggg
gaaggggact aaatctgagc cctctggatc ccttgagaga gttagccaag ggtggccgat ggcaggaggt gaggaccgga
agaagagcgg ggtggagaaa ttagaatccg catgtagagc gcgctatcac aggttgctct gcaacctggc agctgattgg
gaaccggctg tgccaaacga tagggaccac acatgctata ggctctgtag atttttgagg agcaaggaag acactatgat
gatgaattca aaccaactaa gcaccgctgt tttccggaga gtctggatgc ggggatatgc aaaggacttt gacttgactt
ctccttttga actttgggac taagcaaatc ttttagccct gtgggctgtc cctctgtccc attgtaacag tctgtctctg
Forward primer Reporter probe
ctatttcgcc atcatcccac ccggagctgg aggaatgtga tgacgcaggt gcaggtgcag gtgcaggtat gaaactctgt
Reverse primer
tcctggagct agctgactgt cactttgaga gcacagtggc TTGGGCCCCT GCAGCCTAAG AGTACTGGAG
GCTATTCCTG GAGTCTTCAG AGTCCTGAGT CCAGCGAGTC TGGGTGATCC TGGAACTCAG
TGAACTCTGA CTTGGGGTGT CCCTTTAGGG CAGGCTACAG GTCAGGGCCC TTGGTGCACA
GTGACACTAC TGTGAAACCA CCCCAATCCG CTCTCCGGGT TTACTCCGGT CCCGGGCGCG
CACGGGCAGG AGGCCGGGAG CTTGGAACGG GAGGAGCAGC CCGGGGCGGG CTGCCAGGGC
GAGCAAGCGA GCGAGGCGGG GGCGGCGGGT GCAGAGCTAC GGGCGGCGGC GCCCGCGGGT
CACTCTCAGG CCGGGCAGCA GCGACAGGGC GCAGGGTAGC AGGGCGCACC ATG

Supplementary Figure S8. **E-box–like elements in the promoter region of mouse *OCT3***

Several E-box–like elements (CANNTG or CANNTT, shown in green) are present in the promoter region of mouse *OCT3*. CLOCK/BMAL1-mediated circadian control of transcription is driven by non-canonical E-box sequences in the promoter region of the target gene. The first exon sequence is shown in blue and the translation start site (ATG) indicates pink. Forward primer, reporter probe and reverse primer sites used for the ChIP assay (*OCT3*) are shown in red, orange and purple.

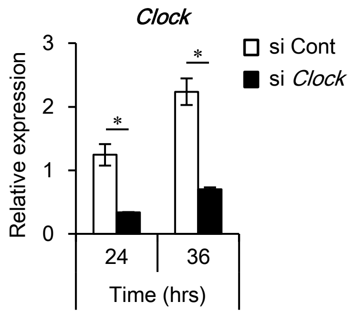

Supplementary Figure S9. Knockdown of *Clock* mRNA using siRNA in wild-type BMMCs
Wild-type BMMCs were transfected with *Clock* siRNAs. *Clock* mRNA expression levels were evaluated 24 and 36 hours after the *Clock* siRNAs transfection. (n = 4)
Unpaired student's t-test; Values represent means ± SD. *$p < 0.05$.
Similar results were obtained at least from 2 independent experiments.

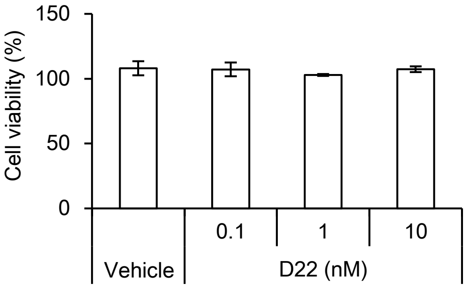

Supplementary Figure S10. D22 does not affect the viability of BMMCs
Wild-type BMMCs (1 x 10^5 cells/well) were cultured with or without the indicated concentrations of D22 for 24 hours in a 96-well microtiter plate. The cell viability was then determined by trypan blue dye exclusion. (n = 3) One-way ANOVA with Bonferroni's test; Values represent means $\pm$ SD. $^*p < 0.05$, $^{**}p < 0.01$.
Similar results were obtained at least from 2 independent experiments.

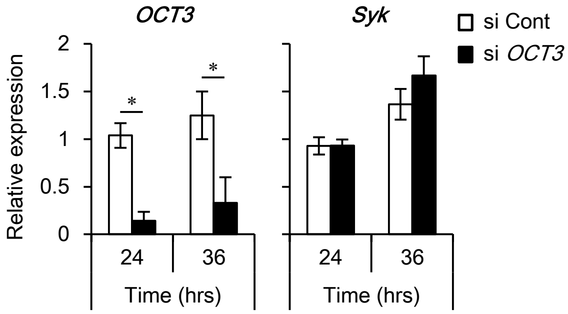

Supplementary Figure S11. **Knockdown of *OCT3* mRNA using siRNA in wild-type BMMCs**
Wild-type BMMCs were transfected with *OCT3* siRNAs. *OCT-3* and *Syk* mRNA expression levels were evaluated 24 and 36 hours after the *OCT3* siRNAs transfection. (n = 4)
Unpaired student's t-test; Values represent means ± SD. *$p < 0.05$.
Similar results were obtained at least from 2 independent experiments.

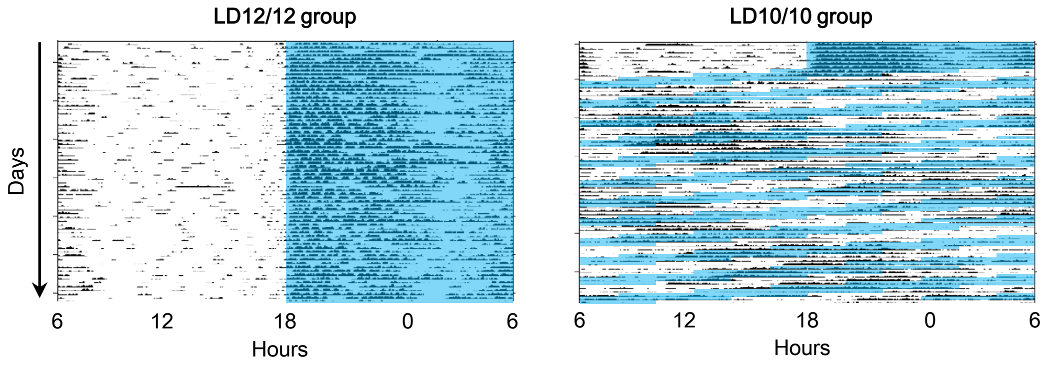

Supplementary Figure S12. **Locomotor activity of mice housed under a 10-hour light / 10-hour dark (LD10/10) cycle or a 12-hour light / 12-hour dark (LD12/12)**

Representative single-plotted actogram showing the locomotor activity of the wild-type mice housed under a LD10/10 cycle (right) or a normal LD12/12 cycle (left). Locomotor activity was recorded as indicated by black dots over a 60-day period. In mice housed under a LD10/10 cycle, mice were kept under a consistent LD12/12 cycle in the first 7 days, and then were exposed to LD10/10 cycles for 60 days. Dark phase is indicated by blue shadow on each record. Please note the locomotor activity became free-running in mice kept under LD10/10 cycle.

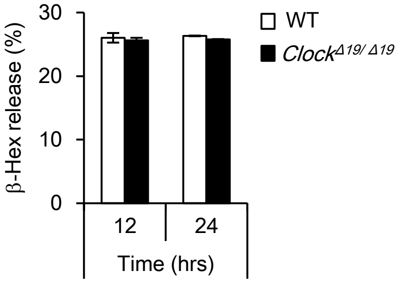

Supplementary Figure S13. **Compound 48/80–induced histamine levels are comparable between wild-type BMMCs cultured for 12 or 24 hours**

Wild-type or *Clock*$^{Δ19/Δ19}$ BMMCs cultured for 12 or 24 hours following a media change for synchronization were stimulated with compound 48/80. Culture supernatants were collected 12 hours after stimulation, and then the levels of compound 48/80–induced β-hexosaminidase release were compared. (n = 6)

One-way ANOVA with Bonferroni's test; Values represent means ± SD. *$p < 0.05$, **$p < 0.01$. Similar results were obtained at least from 2 independent experiments.

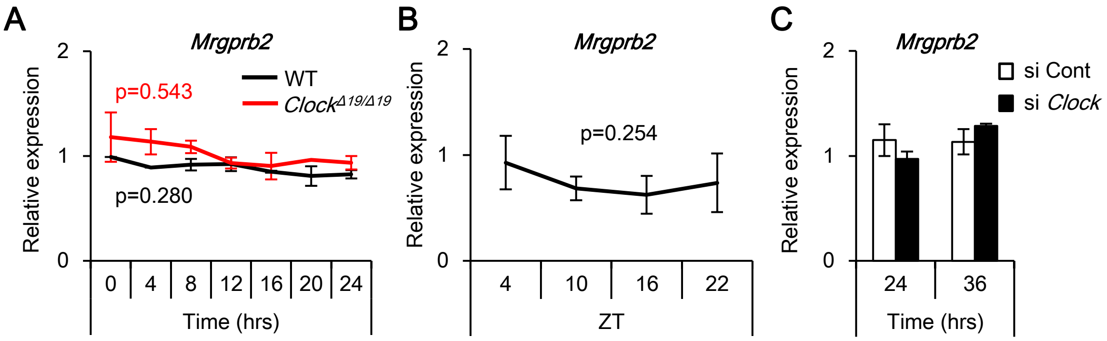

Supplementary Figure S14. **Kinetics of *Mrgprb2* mRNA expression in wild-type BMMCs and in the mouse skin**

A. Wild-type (WT) BMMCs were cultured after a media change for synchronization; at the indicated time points, mRNA was extracted, and *Mrgprb2* mRNA was quantitated by qPCR. (n = 4 per group) P values are shown for one-way ANOVA; Values represent means ± SD.

B. Kinetics of *Mrgprb2* mRNA expression in the skin (ear) of wild-type mice. (n = 4) P values are shown for one-way ANOVA; Values represent means ± SD.

C. Knockdown of *Clock* mRNA using siRNA does not affect *Mrgprb2* mRNA expression in wild-type BMMCs. Wild-type BMMCs were transfected with *Clock* siRNAs as described in Fig. S9. *Mrgprb2* mRNA expression levels were evaluated 24 and 36 hours after the *Clock* siRNAs transfection. (n = 4) Unpaired student's t-test; Values represent means ± SD. *p < 0.05.

Similar results (**A-C**) were obtained at least from 2 independent experiments.

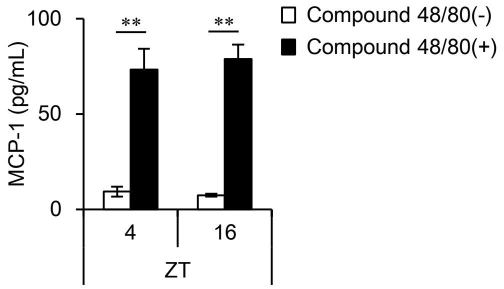

Supplementary Figure S15. Serum monocyte chemoattractant protein (MCP)-1 [CCL2] levels in wild-type mice treated with compound 48/80

Wild-type mice were subcutaneously treated with compound 48/80 at ZT4 or ZT16 and serum samples were collected 30 minutes after the treatment. MCP-1 (CCL2) levels in the serum were measured by ELISA. (n = 8). One-way ANOVA with Bonferroni's test; Values represent means ± SD. *p < 0.05, **p < 0.01.
Similar results were obtained at least from 2 independent experiments.
